# Over-expression of long noncoding RNA BANCR inhibits malignant phenotypes of human bladder cancer

**DOI:** 10.1186/s13046-016-0397-9

**Published:** 2016-08-11

**Authors:** Anbang He, Yuchen Liu, Zhicong Chen, Jianfa Li, Mingwei Chen, Li Liu, Xinhui Liao, Zhaojie Lv, Yonghao Zhan, Chengle Zhuang, Junhao Lin, Weiren Huang, Hongbing Mei

**Affiliations:** 1 Shenzhen Second People’s Hospital, Clinical Medicine College of Anhui Medical University, Shenzhen, 518039 Guangdong China; 2 Key Laboratory of Medical Reprogramming Technology, Shenzhen Second People’s Hospital, The First Affiliated Hospital of Shenzhen University, Shenzhen, 518035 China; 3 Shantou University Medical College, Shantou, 515041 China

**Keywords:** BANCR, Bladder cancer, LncRNAs, Therapeutic target

## Abstract

**Background:**

Accumulating evidences indicated that lncRNAs play crucial regulatory roles in oncogenesis and progression of cancers. BRAF activated non-coding RNA (BANCR) has been identified to contribute to the progression of some human cancers. However, the relationship between BANCR and bladder cancer (BC) is largely unclear.

**Methods:**

BANCR expression levels in BC, paired non-cancer tissues and BC cell lines were detected by real-time quantitative RT-PCR (qRT-PCR). The relationships between BANCR expression levels and the clinical characteristics were evaluated. BANCR expression was enhanced by transfecting a pcDNA-BANCR vector. We used both CCK-8 assay and Edu assay to detect cell proliferation. We also detect cell apoptosis and migration by using ELISA assay, Flow cytometry and transwell assay, respectively. All statistical analyses were executed by using the SPSS 20.0 software.

**Results:**

BANCR expression levels were remarkably decreased in BC tissues compared with adjacent noncancerous tissues. BANCR expression levels in two BC cell lines were also significantly down-regulated. Clinicopathologic analysis revealed that low BANCR expression was positively correlated with TNM stage, but not associated with other clinicopathological characteristics. BANCR has been successfully overexpressed in BC cell lines (T24 and SW780) by transfecting a pcDNA-BANCR vector. Cell proliferation inhibition, apoptosis induction and migration suppression were also observed in pCDNA-BANCR-transfected T24 and SW780 cells.

**Conclusions:**

These data suggested that BANCR represents a tumor suppressor player in bladder cancer, contributes to tumor proliferation, apoptosis and migration, and may serve as a new candidate biomarker and a potential therapeutic target for patients with BC.

## Background

Bladder cancer (BC) is one of the most common malignancies over the world, and its incidence and mortality have captured great attention in the past decades [1, 2]. Although a variety of treatments are available for patients with bladder cancer, such as surgery, chemotherapy and radiation therapy, but the 5-year cancer-specific survival rate remains frustrated [3–5]. Lack of sophisticated understanding of pathogenetic mechanism is one of the most crucial reasons for high case fatality rate of bladder cancer. So, revealing the pathogenetic mechanism of bladder cancer is indispensable for developing an effective diagnosis or treatment.

With the rapid development of second-generation sequencing technology, a multitude of long noncoding RNAs (lncRNAs) have been found to be dysregulated and involved in the development of various human diseases, particularly in cancers [6–8]. Accumulating evidences demonstrated that lncRNAs play important regulatory roles in diverse cellular processes, such as regulation of gene expression, posttranslational processing and tumorigenesis [7, 9]. BRAF activated non-coding RNA (BANCR), a 693-nt lncRNA encoded on human chromosome 9, has been found to be aberrant expressed in quite a few cancers, such as colorectal cancer [10], retinoblastoma [11], melanoma [12], papillary thyroid carcinoma [13], lung carcinoma [14],gastric cancer [15] and hepatocellular carcinoma [16]. However, the significance of lncRNA BANCR in bladder cancer is completely unknown.

Thus, in the present research, we identified the clinical significance of lncRNA BANCR in 54 clinical bladder cancer samples and investigated the effects of BANCR expression on bladder cancer cells in vitro. Moreover, further experiments indicated that the overexpression of lncRNA BANCR could inhibit proliferation, induce apoptosis and suppress migration of the bladder cancer cell lines.

## Methods

### Patient samples

In this research, a total of 54 patients with urothelial neoplasms of the bladder who received radical cystectomy were included. Bladder cancer tissues and their pair-matched adjacent normal tissues were snap-frozen in liquid nitrogen quickly after resection.

### Cell lines and cell culture

Human bladder cancer cell lines (T24, SW780) and SV-40-immortalized human uroepithelial cell line (SV-HUC-1) were purchased from the Institute of Cell Biology, Chinese Academy of Sciences (Shanghai, China). The T24 and SW780 cells were cultured in Dulbecco’s Modified Eagle Medium (Invitrogen, Carlsbad, CA, USA) plus 1 % antibiotics (100U/ml penicillin and100 μg/ml streptomycin sulfates) and 10 % fetal bovine serum (FBS). The SV-HUC-1 cells were cultured in F12K (Invitrogen, Carlsbad, CA, USA) plus 1 % antibiotics (100U/ml penicillin and100 μg/ml streptomycin sulfates) and 10 % fetal bovine serum (FBS). All cells were cultured at 37 °C, in a humidified atmosphere with 5 % CO_2_.

### Plasmid DNA Transfection

The BANCR sequence and negative control were synthesized and subcloned into pCDNA3.1 (GenePharma, Suzhou, China) vector. The pCDNA-BANCR or negative control was transfected into T24 and SW780 cells cultured in six-well plates using Lipofectamine 3000 Transfection Reagent (Invitrogen, Carlsbad, CA, USA) according to the manufacturer’s protocol. The expression level of BANCR was detected by qRT-PCR.

### RNA extraction and qRT-PCR

All total RNA was extracted from bladder cancer tissues or cells after transfection by using the Trizol reagent (Invitrogen, Carlsbad, CA, USA) according to the manufacturer’s protocol. Qualified total RNA was then reverse transcribed to cDNA by utilizing Prime Script RT Reagent Kit with gDNA Eraser (Takara, Dalian, China) following the manufacturer’s instructions. The quantitative real-time polymerase chain reaction (qRT-PCR) was executed using SYBR Green PCR kit (Takara, Dalian, China) following the manufacturer’s instructions. Glyceraldehyde 3-phosphate dehydrogenase (GAPDH) was measured as an internal control. The primer sequences were as follows: BANCR primers: 5’- ACAGGACTCCATGGCAAACG-3’ (forward) and 5’- ATGAAGAAAGCCTGGTGCAGT-3’ (reverse), and GAPDH primers: 5’-CGCTCTCTGCTCCTCCTGTTC-3’ (forward), 5’–ATCCGTTGACTCCGACCTTCAC-3’ (reverse). The reactions were carried out on an ABI PRISM 7300 Fluorescent Quantitative PCR System (Applied Biosystems, Foster City, CA, USA) in triplicate. The average value in each triplicate was used to calculate the relative amount of BANCR using 2^−ΔΔCt^ methods.

### Cell proliferation assays

Cell proliferation was determined by using Cell Counting Kit-8, CCK-8 (Beyotime Institute of Biotechnology, Shanghai, China) and 5-ethynyl-20-deoxyuridine (Edu) assay kit (Ribobio, Guangzhou, China), respectively, according to the manufacturer’s instructions. For CCK-8 assay, 5 × 10^3^cells/ well were seeded in a 96-well plate for 24 h, then transiently transfected with pCDNA-BANCR or negative control. CCK-8 assay was performed according to the previous study. Edu incorporation assay was carried out according to previous studies [17]. All experiments were performed at least three times.

### Caspase-3 ELISA assay

Bladder cancer cells T24 and SW780 were transfected with pCDNA-BANCR or negative control. At 48 h after transfection, the activity of caspase 3 was detected by using the Caspase-3 Colorimetric Assay kit (Abcam, Cambridge, UK) according to the manufacturer’s protocol. Each experiment was performed in triplicate.

### Flow cytometry assay

T24 and SW780 cells were transfected with pCDNA-BANCR or negative control in normal medium. After 48 h, cells were harvested for flow cytometry assay. After double staining with FITC-Annexin V and PI according to the manufacturer’s instructions, cell apoptosis was determined by using flow cytometry (EPICS, XL-4, Beckman, CA, USA).

### Transwell assay

The cell motility assay were performed using a transwell insert (8 μm, Corning). After 24 h, 5x10^4^ cells transfected with pCDNA-BANCR or negative control were first starved in 200 ml serumfree medium and then placed in the uncoated dishes. Transwell assay was performed according to one previous study [18].

### Statistical analyses

All statistical analyses were executed by using SPSS 20.0 software (IBM, Chicago, IL, USA). Paired samples’t test was used to analyze the BANCR expression difference between bladder cancer tissues and para-cancer tissues. CCK-8 assay data were analyzed by ANOVA and independent samples’ *t* test was utilized to analyze other data. The chi-square test was used to exam the relationship between BANCR expression level and clinicopathologic characteristics. Differences were considered statistically significant at *p* < 0.05.

## Results

### BANCR was down-regulated in bladder cancer tissues and cells

**Fig. 1:**
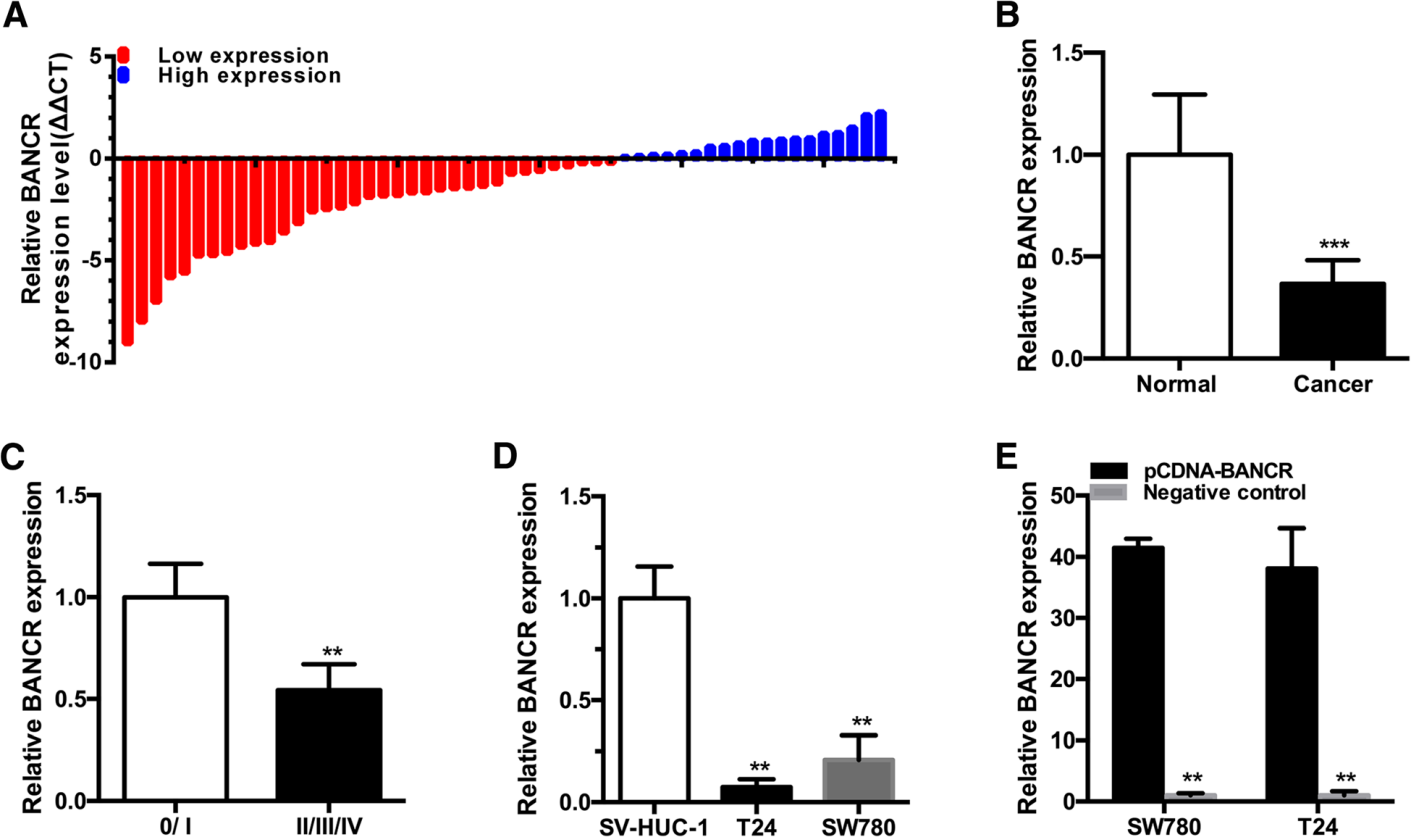
The long noncoding RNA BANCR was down-regulated in bladder cancer. The relative expression levels of BANCR were determined using RT- qPCR. **a** BANCR expression levels were lower in bladder cancer tissues. **b** The relative expression level of BANCR was significantly lower in bladder cancer tissues compared with pair-matched adjacent normal bladder tissues. **c** BANCR expression levels were dramatically lower in patients with higher TNM stage. **d** BANCR expression levels were lower in bladder cancer T24 and SW780 cells than those in SV-40-immortalized human uroepithelial cells. **e** The pCDNA-BANCR significantly up-regulated the expression level of BANCR in SW780 and T24 cells. Data (**b** and **c**) are shown as mean ± SEM. Data (**d** and **e**) are shown as mean ± SD. (**P < 0.01, ***P < 0.001)

The relative expression level of BANCR was measured by using qRT-PCR in bladder cancer tissues and pair-matched adjacent normal bladder tissues from 54 bladder cancer patients. Compared with pair-matched adjacent normal bladder tissues, the BANCR expression was down-regulated significantly in 64.8 % (35 of 54) of cancer tissues (Fig. 1a and b, ***P < 0.001). Moreover, down-regulated BANCR expression was significantly correlated with advanced TNM stage (Fig. 1c, **P < 0.01). Compared with the SV-HUC-1 cell line, the BANCR expression was decreased significantly in bladder cancer cellsT24 and SW780 (Fig. 1d, **P < 0.01). These data demonstrated that BANCR may function as a tumor suppressor gene in bladder cancer. Patients’ clinical parameters are listed in Table 1.

**Table 1:** Correlation between BANCR expression and clinicopathological characteristics of bladder cancer patients

Parameters	Group	Total	BANCR expression		P value
			High	Low	
Gender	Male	38	16	22	0.09
	Female	16	3	13	
Age(years)	<65	29	12	17	0.230
	≥65	25	7	18	
Tumor size (cm)	<3	24	11	13	0.065
	≥3	30	8	22	
Histological grade	L	20	9	11	0.194
	H	34	10	24	
TNM stage	0/ I	15	10	5	0.004*
	II/III/IV	39	9	30	
Lymph nodes metastasis	N0	50	18	32	0.559
	N1 or above	4	1	3	

*P < 0.05 was considered significant (Chi-square test between 2 groups)

### Specific plasmid vectors up-regulated the expression of BANCR

Bladder cancer cell lines T24 and SW780 were cultured and transfected with pCDNA-BANCR or negative control. At 48 h after transfection, the related expression level of BANCR was analyzed by qRT-PCR and the results showed that the relative expression level of BANCR in T24 and SW780 (Fig. 1e, **P < 0.01) cells was significantly up-regulated.

### Overexpression of BANCR inhibited cell proliferation

**Fig. 2:**
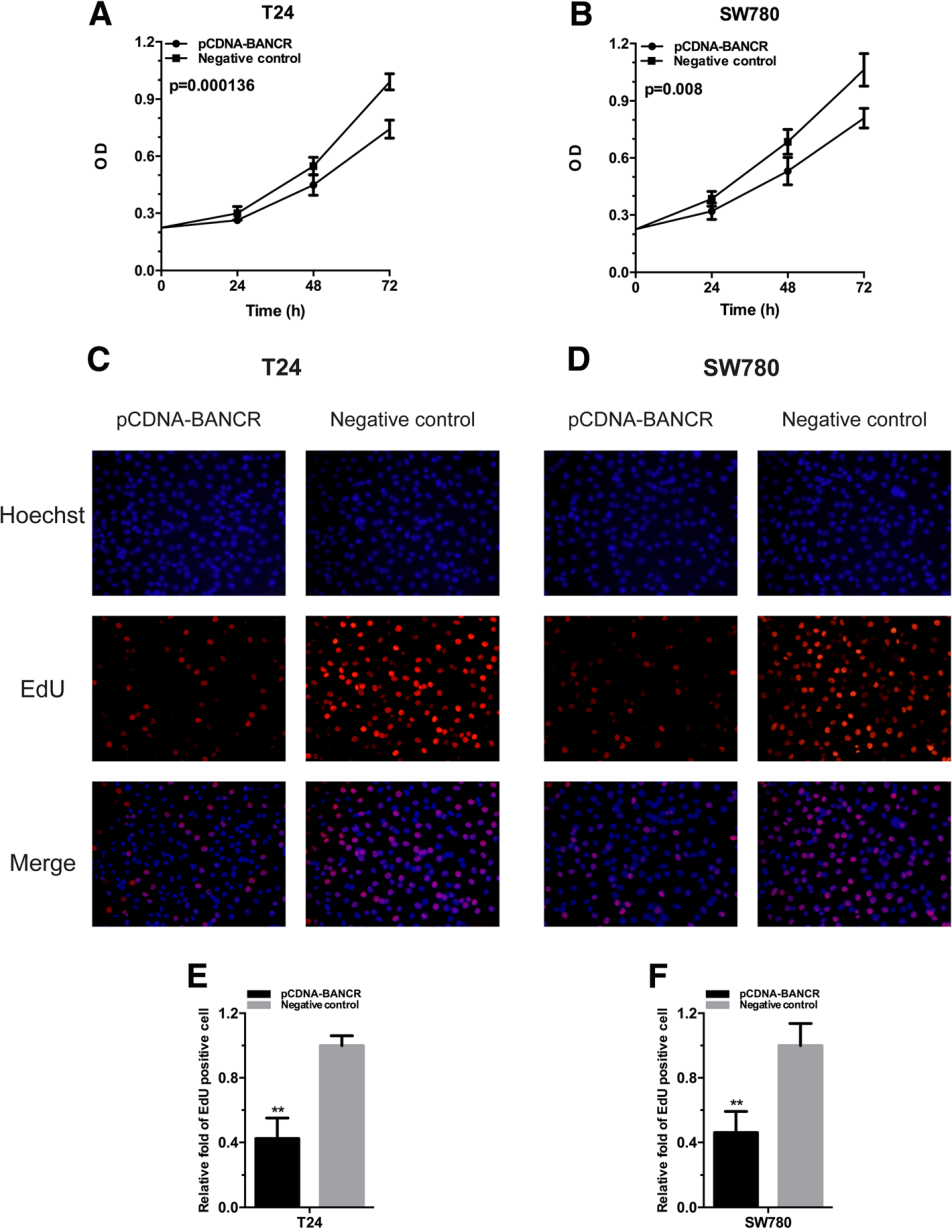
Overexpression of lncRNA BANCR inhibited cell proliferation in bladder cancer cells. Cell proliferation was detected by both CCK-8 assay and Edu assay. Cell proliferation inhibition was observed in bladder cancer T24 (**a**, **c** and **e**), SW780 (**b**, **d** and **f**). Data are shown as mean ± SD. (**P < 0.01, ***P < 0.001)

To explore the possible impact of BANCR on the growth of bladder cancer cells, the cell proliferation activities of T24 and SW780 were determined by both CCK-8 assay and Edu assay. Compared with the negative control group, cell growth arrest was observed in bladder cancer cells T24 (Fig. 2a, c and e) and SW780 (Fig. 2b, d and f) which were cultured and transfected with pCDNA-BANCR. The results suggested that BANCR inhibits cell proliferation in bladder cancer cells.

### Overexpression of BANCR induced apoptosis

**Fig. 3:**
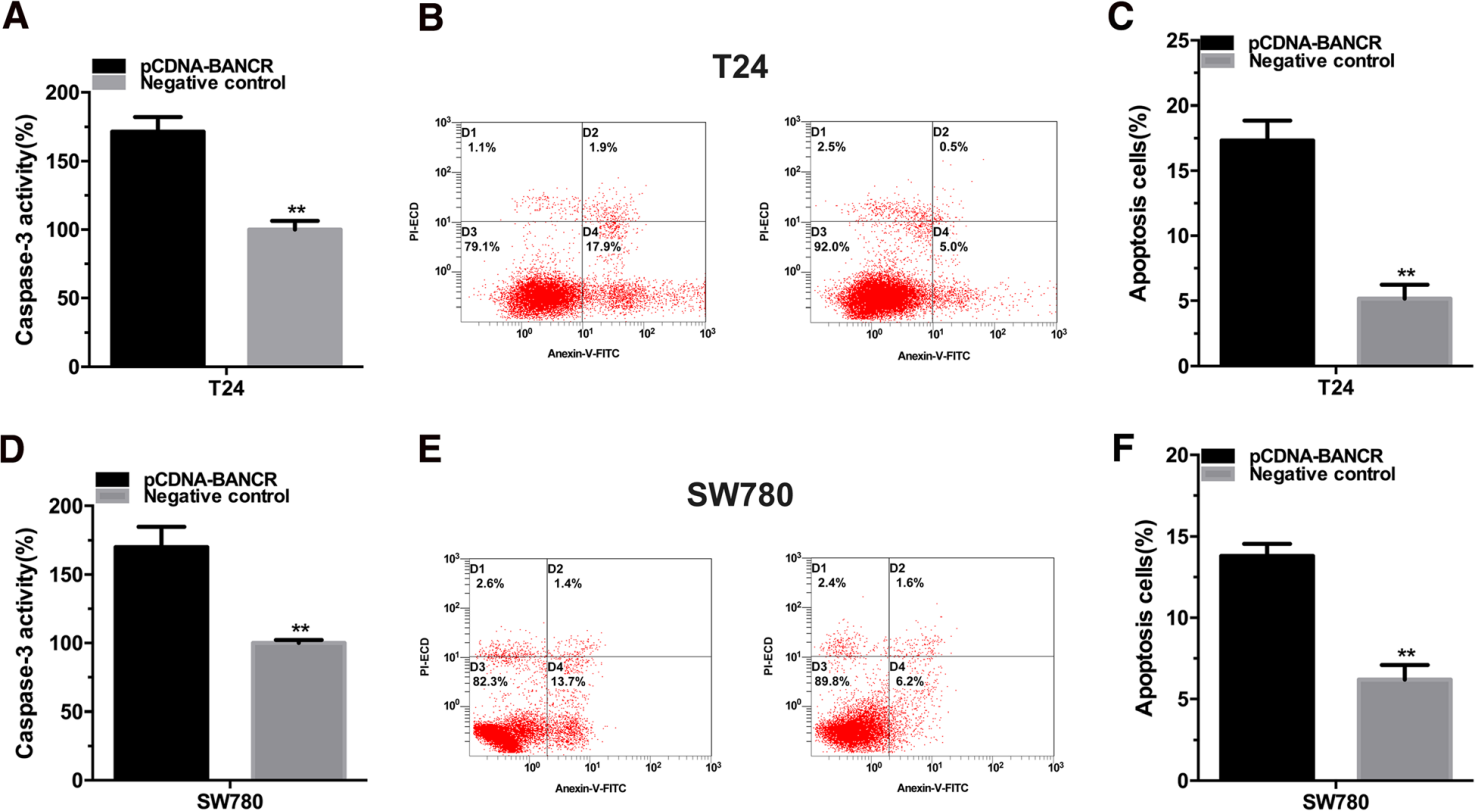
Overexpression of lncRNA BANCR induced cell apoptosis in bladder cancer cells. Cell apoptosis was determined by both ELISA assay and Flow cytometry. Induced cell apoptosis was observed in bladder cancer T24 (**a**, **b** and **c**) and SE780 (**d**, **e** and **f**) cells. Data are shown as mean ± SD. (**P < 0.01, ***P < 0.001)

Furthermore, we investigated whether BANCR could induce cell apoptosis in bladder cancer. Bladder cancerT24 and SW780 cells were transfected with pCDNA-BANCR or negative control. The relative activity of caspase-3 was determined using the caspase 3 enzyme-linked immunosorbent assay (ELISA) (Fig. 3a, d) and the apoptosis ratio in bladder cancer cells was measured using flow cytometry assay. (Figure 3b, c, e and f). Induced cell apoptosis was observed in both bladder cancer and SW780 cell lines. These results indicated that BANCR induces cell apoptosis in bladder cancer.

### Overexpression of BANCR inhibited cell migration

**Fig. 4:**
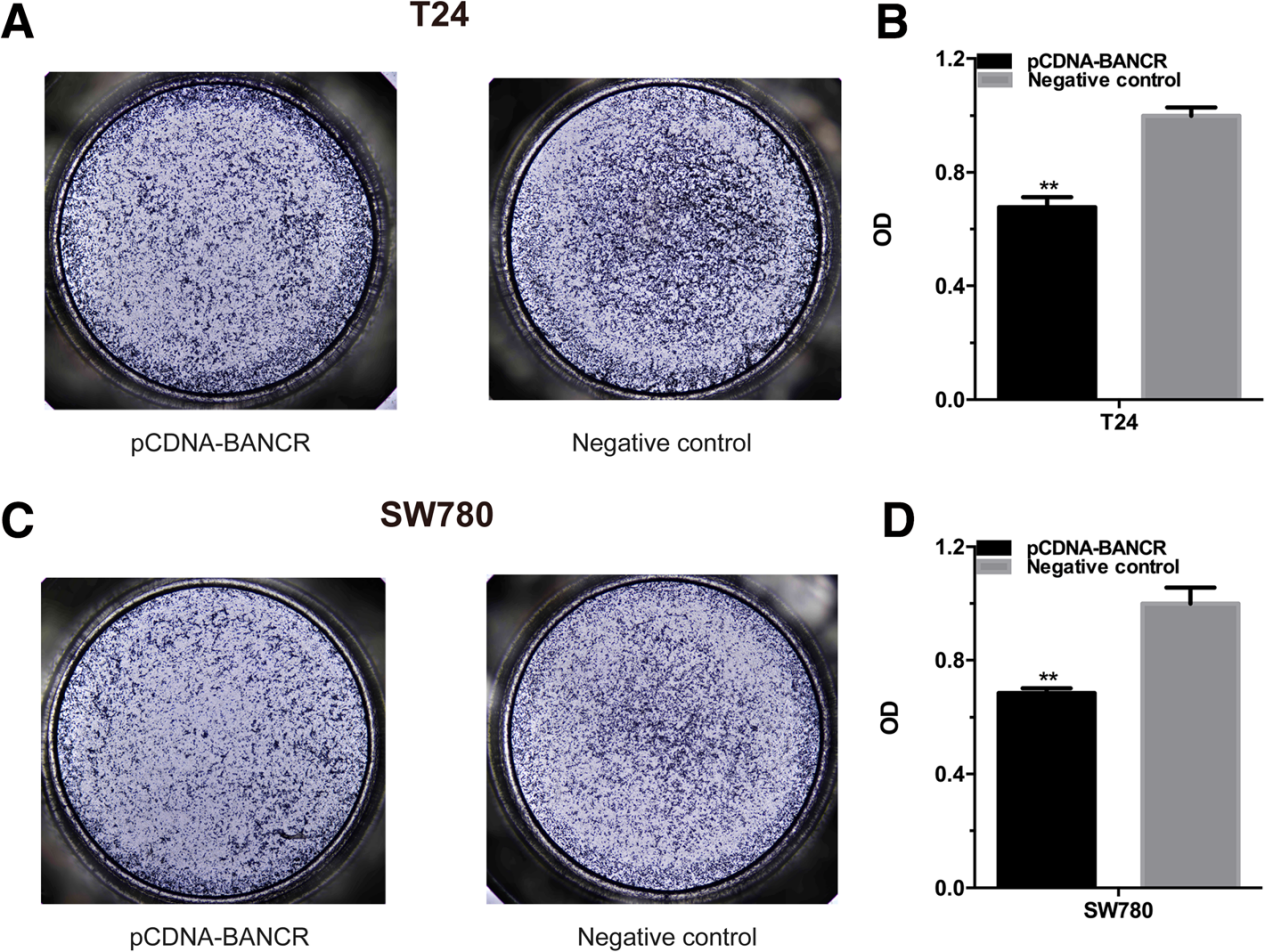
overexpression of lncRNA BANCR inhibited cell migration in bladder cancer cells. Cell migration was detected by transwell assay. Cell migration inhibition was observed in bladder cancer T24 (**a** and **b**) and SW780 (**c** and **d**) cells. Data are shown as mean ± SD. (**P < 0.01, ***P < 0.001)

Lastly, we further determined whether BANCR suppressed cell migration in bladder cancer. The cell migration activities of bladder cancer T24 and SW780 cells transfected with pCDNA-BANCR or negative control were determined by transwell assay. Cell migration arrest was observed in T24 (Fig. 4a, b), SW780 (Fig. 4c, d) as expected. These results manifested that BANCR inhibits cell migration in bladder cancer.

## Discussion

Until now, lots of works indicate that lncRNAs play vital regulatory roles in tumorigenesis and tumor progression and act as potential oncogenes or tumor suppressor genes [19, 20]. Recently, several lncRNAs have been found to play an emerging role in various cancers, contributing to tumor proliferation, apoptosis, and survival, such as PANDAR [18], MEG3 [21].

BANCR, firstly found by Flockhart RJ et al. [22], has been discovered to be up-regulated in some kinds of tumors, including retinoblastoma, melanoma, papillary thyroid carcinoma, gastric cancer and hepatocellular carcinoma. In contrast, decreased expression of BANCR has been found in both colorectal and lung cancers. BANCR promotes cell proliferation by regulating MAPK pathway activation in both malignant melanoma and lung carcinoma [14, 23]. Besides, downregulation of BANCR promotes colorectal cancer cell proliferation through decreasing expression of p21 protein [10]. Furthermore, BANCR expression promoted non-small cell lung cancer cell migration and invasion through regulating E-cadherin, N-cadherin and Vimentin expression, all of which play crucial roles in epithelial-mesenchymal transition (EMT) [24]. These evidences suggest that BANCR may be involved in different signaling pathways and plays its unique role in each cancer. However, the functions of BANCR in bladder cancer were completely unknown.

To the best of our knowledge, this is the first evidence that BANCR was significantly downregulated in bladder cancer tissues compared with adjacent noncancerous tissues, and low BANCR expression in bladder cancer patients was positively correlated with advanced TNM stage. Furthermore, compared with the SV-HUC-1 cell line, the BANCR expression in bladder cancer cell lines (T24, SW780) was also obviously downregulated. These results manifest that lncRNA BANCR may emerge as a novel player in bladder cancer. To further explore the biological functions of lncRNA BANCR, we detected the cell proliferation, apoptosis and migration by overexpressing BANCR in bladder cancer T24 and SW780 cells. pCDNA-BANCR-mediated overexpression of BANCR significantly inhibited proliferation, promoted apoptosis and suppressed metastasis capabilities of bladder cancer T24 and SW780 cells compared with control group, suggesting that up-regulated BANCR expression could suppress the progression and development of bladder cancer.

## Conclusion

In conclusion, the present study suggested that lncRNA BANCR was decreased in BC tissues and correlated with advanced TNM stage. Moreover, we preliminarily revealed the significant function of BANCR in regulating cell proliferation, apoptosis, and migration of BC cells. These findings indicated that BANCR may emerge as a tumor suppressor gene and could become a novel promising candidate for the prognosis and therapy for BC in the future. However, the prognostic role of BANCR should be verified in a larger case series, composed by firstly diagnosed patients, treated with TURB instead of cystectomy and with a minimum of 3 years of follow up. Moreover, the molecular mechanism by which BANCR was decreased in bladder cancer also should be investigated in the future works.

## References

[1] Siegel RL, Miller KD, Jemal A (2016). Cancer statistics, 2016. CA Cancer J Clin.

[2] Jacobs BL, Lee CT, Montie JE (2010). Bladder cancer in 2010: How far have we come?. CA Cancer J Clin.

[3] Racioppi M, D’Agostino D, Totaro A, Pinto F, Sacco E, D’Addessi A, Marangi F, Palermo G, Bassi PF (2012). Value of current chemotherapy and surgery in advanced and metastatic bladder cancer. Urol Int.

[4] Sofra M, Fei PC, Fabrizi L, Marcelli ME, Claroni C, Gallucci M, Ensoli F, Forastiere E (2013). Immunomodulatory effects of total intravenous and balanced inhalation anesthesia in patients with bladder cancer undergoing elective radical cystectomy: preliminary results. J Exp Clin Cancer Res.

[5] Rose TL, Milowsky MI (2016). Improving systemic chemotherapy for bladder cancer. Curr Oncol Rep.

[6] Ponting CP, Oliver PL, Reik W (2009). Evolution and functions of long noncoding rnas. Cell.

[7] Cheetham SW, Gruhl F, Mattick JS, Dinger ME (2013). Long noncoding rnas and the genetics of cancer. Br J Cancer.

[8] Geisler S, Coller J (2013). Rna in unexpected places: long non-coding rna functions in diverse cellular contexts. Nat Rev Mol Cell Biol.

[9] Isin M, Dalay N (2015). Lncrnas and neoplasia. Clin Chim Acta.

[10] Shi Y, Liu Y, Wang J, Jie D, Yun T, Li W, Yan L, Wang K, Feng J (2015). Downregulated long noncoding rna bancr promotes the proliferation of colorectal cancer cells via downregualtion of p21 expression. PLoS One.

[11] Su S, Gao J, Wang T, Wang J, Li H, Wang Z (2015). Long non-coding rna bancr regulates growth and metastasis and is associated with poor prognosis in retinoblastoma. Tumour Biol.

[12] Li R, Zhang L, Jia L, Duan Y, Li Y, Bao L, Sha N (2014). Long non-coding rna bancr promotes proliferation in malignant melanoma by regulating mapk pathway activation. PLoS One.

[13] Wang Y, Guo Q, Zhao Y, Chen J, Wang S, Hu J, Sun Y (2014). Braf-activated long non-coding rna contributes to cell proliferation and activates autophagy in papillary thyroid carcinoma. Oncol Lett.

[14] Jiang W, Zhang D, Xu B, Wu Z, Liu S, Zhang L, Tian Y, Han X, Tian D (2015). Long non-coding rna bancr promotes proliferation and migration of lung carcinoma via mapk pathways. Biomed Pharmacother.

[15] Zhang ZX, Liu ZQ, Jiang B, Lu XY, Ning XF, Yuan CT, Wang AL (2015). Braf activated non-coding rna (bancr) promoting gastric cancer cells proliferation via regulation of nf-kappab1. Biochem Biophys Res Commun.

[16] Zhou T, Gao Y (2016). Increased expression of lncrna bancr and its prognostic significance in human hepatocellular carcinoma. World J Surg Oncol.

[17] Zhan Y, Liu Y, Wang C, Lin J, Chen M, Chen X, Zhuang C, Liu L, Xu W, Zhou Q, Sun X, Zhang Q, Zhao G, Huang W. Increased expression of sumo1p3 predicts poor prognosis and promotes tumor growth and metastasis in bladder cancer. Oncotarget. 2016. 10.18632/oncotarget.6946 PMC4941296 26799188

[18] Zhan Y, Lin J, Liu Y, Chen M, Chen X, Zhuang C, Liu L, Xu W, Chen Z, He A, Zhang Q, Sun X, Zhao G, Huang W (2016). Up-regulation of long non-coding rna pandar is associated with poor prognosis and promotes tumorigenesis in bladder cancer. J Exp Clin Cancer Res.

[19] Wu J, Zhang J, Shen B, Yin K, Xu J, Gao W, Zhang L (2015). Long noncoding rna lnctcf7, induced by il-6/stat3 transactivation, promotes hepatocellular carcinoma aggressiveness through epithelial-mesenchymal transition. J Exp Clin Cancer Res.

[20] Martens-Uzunova ES, Bottcher R, Croce CM, Jenster G, Visakorpi T, Calin GA (2014). Long noncoding rna in prostate, bladder, and kidney cancer. Eur Urol.

[21] Peng W, Si S, Zhang Q, Li C, Zhao F, Wang F, Yu J, Ma R (2015). Long non-coding rna meg3 functions as a competing endogenous rna to regulate gastric cancer progression. J Exp Clin Cancer Res.

[22] Flockhart RJ, Webster DE, Qu K, Mascarenhas N, Kovalski J, Kretz M, Khavari PA (2012). Brafv600e remodels the melanocyte transcriptome and induces bancr to regulate melanoma cell migration. Genome Res.

[23] Staff PO (2015). Correction: long non-coding rna bancr promotes proliferation in malignant melanoma by regulating mapk pathway activation. PLoS One.

[24] Sun M, Liu XH, Wang KM, Nie FQ, Kong R, Yang JS, Xia R, Xu TP, Jin FY, Liu ZJ, Chen JF, Zhang EB, De W, Wang ZX (2014). Downregulation of braf activated non-coding rna is associated with poor prognosis for non-small cell lung cancer and promotes metastasis by affecting epithelial-mesenchymal transition. Mol Cancer.

